# Association between vitamin D deficiency and longitudinal risk of head and neck cancer: a multi-institutional retrospective study

**DOI:** 10.3389/fnut.2026.1826071

**Published:** 2026-07-01

**Authors:** I-Wen Chen, Hsiu-Lan Weng, Yu-Li Pang, Chien-Ming Lin, Yi-Chen Lai, Kuo-Chuan Hung

**Affiliations:** 1 Department of Anesthesiology, Chi Mei Hospital, Liouying, Tainan, Taiwan; 2 Department of Anesthesiology, E-Da Hospital, I-Shou University, Kaohsiung, Taiwan; 3 Department of Anesthesiology, Chi Mei Medical Center, Tainan, Taiwan

**Keywords:** dose–response, head and neck cancer, laryngeal cancer, propensity score matching, retrospective cohort study, vitamin D deficiency

## Abstract

**Background:**

Vitamin D is implicated in cancer biology through its regulation of cellular proliferation, apoptosis, and immune surveillance; however, evidence linking vitamin D deficiency (VDD) to head and neck cancer (HNC) risk remains limited by small sample sizes and inadequate control for confounders.

**Methods:**

Using the TriNetX Global Collaborative Network, we conducted a retrospective cohort study comparing adults with laboratory-confirmed VDD (25-hydroxyvitamin D < 20 ng/mL) against controls (≥30 ng/mL). After applying the exclusion criteria and 1:1 propensity score matching, 108,010 patients per group were retained. In a separate exploratory dose–response analysis, patients with vitamin D insufficiency (20.0–29.9 ng/mL) were compared with the same sufficiency threshold (≥30 ng/mL). A 180-day landmark period was applied to mitigate reverse causation, with follow-up extending to 10 years. The primary outcome was incident HNC; the secondary outcomes included laryngeal, oral, and other HNC subtypes. Positive (osteoporotic fracture) and negative (appendicitis) control outcomes were assessed to evaluate internal validity.

**Results:**

VDD was associated with a significantly higher risk of incident HNC (HR 1.56, 95% CI 1.43–1.70, *p* < 0.001). Consistent associations were observed across anatomical subtypes: laryngeal cancer (HR 1.72, *p* < 0.001), oral cancer (HR 1.53, *p* < 0.001), and other HNCs (HR 1.46, *p* < 0.001). Vitamin D insufficiency was examined separately in 140,247 matched pairs and was associated with a smaller increase in HNC risk than VDD (HR 1.21, *p* < 0.001), supporting a dose–response pattern. Control outcomes validated the study design, with osteoporotic fracture confirming the expected positive association (HR 1.58, *p* < 0.001) and appendicitis demonstrating no spurious signal (HR 1.06, *p* = 0.226). The association was prominent in patients aged >50 years but absent in younger adults (*p* for interaction <0.001). Sensitivity analyses with extended landmark periods and alternative cohort definitions yielded consistent results.

**Conclusion:**

In this observational study, VDD was significantly associated with an increased risk of HNC, with consistency across subtypes and a dose–response gradient. However, the observational design precludes causal inference, and residual confounding cannot be fully excluded. Prospective studies with detailed lifestyle data are needed to confirm these findings.

## 1 Introduction

Head and neck cancer (HNC) represents the seventh most common malignancy worldwide and encompasses a heterogeneous group of tumors arising from the oral cavity, pharynx, larynx, nasal cavity, paranasal sinuses, and salivary glands (1–3). Established risk factors include tobacco use, alcohol consumption, and human papillomavirus infection (4–7); however, these factors do not fully account for disease occurrence, and identifying additional modifiable determinants remains a priority for prevention strategies. Vitamin D, a secosteroid hormone classically recognized for its role in calcium and bone homeostasis, has increasingly been implicated in cancer biology (8–10). Through binding to the vitamin D receptor expressed in a wide range of epithelial tissues, the biologically active metabolite calcitriol regulates cellular proliferation, differentiation, apoptosis, and immune surveillance—processes central to carcinogenesis (9, 11, 12). Epidemiological studies have demonstrated inverse associations between circulating 25-hydroxyvitamin D levels and the risk of several malignancies, including colorectal, breast, and prostate cancers (13–16). However, evidence from randomized controlled trials has been less conclusive. In the large VITAL trial, vitamin D3 supplementation did not significantly reduce the incidence of invasive cancer compared with placebo (17), and updated meta-analyses of randomized trials similarly found no significant reduction in total cancer incidence, although potential effects on cancer mortality have been suggested (18, 19). Given that the upper aerodigestive tract epithelium expresses vitamin D receptors and metabolizing enzymes, a similar protective role in HNC has been hypothesized (20, 21).

However, the existing literature on vitamin D status and HNC risk is characterized by heterogeneity in study design and findings, with limited prospective data, small case numbers, and incomplete adjustment for key confounders, which together hinder definitive interpretation of this association. A systematic review (22) identified only five prospective studies that measured vitamin D levels before cancer diagnosis, with conflicting results: two reported (23, 24) an inverse association and two (25, 26) found no significant relationship. Critically, these studies were limited by small sample sizes (typically fewer than 400 cases), inability to examine individual anatomical subtypes, and insufficient adjustment for confounders, such as smoking, alcohol use, and nutritional status (23, 24). Moreover, most cross-sectional and case–control studies measured vitamin D levels after cancer diagnosis, precluding the assessment of temporal directionality and raising concerns about reverse causation (22).

To address these gaps, we conducted a multi-institutional retrospective cohort study using the TriNetX research network to examine the association between pre-diagnostic vitamin D deficiency (VDD) and the risk of incident HNC. We further evaluated whether this association was consistent across anatomical subtypes and whether a dose–response relationship existed between vitamin D levels and cancer risk.

## 2 Methods

### 2.1 Data sources

This retrospective cohort study was conducted using data from the TriNetX Global Collaborative Network, a federated research platform that aggregates de-identified electronic health records from multiple healthcare organizations across different countries. The network includes data on demographics, diagnoses, procedures, medications, and laboratory measurements, which are standardized and updated in near real time. Data are contributed by participating institutions and remain locally stored, with analyses performed through a secure, distributed query system. Previous studies have described the structure, data quality, and research applications of the TriNetX platform (27–29).

### 2.2 Inclusion and exclusion criteria

The study protocol was reviewed and approved by the Institutional Review Board of Chi Mei Medical Center (IRB number: No. 11310-E04), which waived the requirement for informed consent owing to the use of de-identified data. Adult patients (≥18 years) with at least one recorded serum 25-hydroxyvitamin D measurement between January 1, 2014, and December 31, 2023, were identified from the TriNetX Global Collaborative Network. Patients were categorized according to vitamin D status at the index measurement. The VDD cohort consisted of individuals with a first qualifying serum 25-hydroxyvitamin D level <20 ng/mL (30, 31), whereas the control cohort included patients with levels ≥30 ng/mL. To reduce exposure misclassification, patients in the VDD cohort were required to have no documented 25-hydroxyvitamin D level ≥30 ng/mL during the preceding 5 years, while individuals in the control cohort were required to have no recorded level <20 ng/mL during the same period.

### 2.3 Exclusion criteria

To improve outcome ascertainment and reduce surveillance bias, eligible patients were required to have at least one documented head and neck diagnostic evaluation, such as laryngoscopy, nasopharyngoscopy, imaging studies, or biopsy procedures, during follow-up between 6 months and 10 years after the index date. Patients with conditions indicating acute physiological instability at the time of vitamin D testing were excluded, including pregnancy, severe sepsis, acute kidney failure, or receipt of critical care services within 1 month before vitamin D measurement. To minimize confounding from severe systemic illness, patients with end-stage renal disease, advanced chronic kidney disease (stage 4 or 5), dialysis dependence, or human immunodeficiency virus infection prior to the index date were excluded. Patients who died within 6 months after the index date were also excluded to ensure stable baseline health and sufficient opportunity for outcome assessment.

A landmark analysis approach was applied to further minimize reverse causation. Patients with a diagnosis of head and neck malignancy within 6 months after the index date were excluded to ensure that vitamin D status preceded cancer detection. Follow-up for incident HNC began 180 days after the index date and continued for up to 10 years.

### 2.4 Data collection and propensity score matching

Baseline demographic characteristics, comorbid conditions, laboratory measurements, and medication use were extracted from the TriNetX platform within 5 years preceding the index date. Demographic variables included age, sex, race, and body mass index (BMI). Comorbidity profiles were identified using diagnostic codes and included conditions relevant to HNC risk (Supplementary Table 1). Because direct measures of lifestyle factors are limited in electronic health records, surrogate variables were used: nicotine dependence or chronic obstructive pulmonary disease (COPD) were used as proxies for smoking exposure, whereas alcohol-related disorders and diagnoses of gastritis or duodenitis were used as indicators of alcohol consumption. Gastritis and duodenitis were included as complementary surrogates because alcohol-related disorder codes likely underestimate moderate or hazardous drinking (32); these gastrointestinal conditions may reflect alcohol-related upper gastrointestinal mucosal injury and therefore provide a broader, albeit imperfect, proxy for alcohol exposure (33). Medication exposure was also assessed, with a focus on vitamin D supplementation and antidiabetic therapies. Antidiabetic medications were included to account for metabolic disease severity, given that diabetes is independently associated with both VDD and altered cancer risk, and that certain agents may exert independent effects on cancer risk (34–36).

Propensity score matching was performed to reduce confounding and selection bias. Patients with VDD were matched with controls at a 1:1 ratio using a greedy nearest-neighbor algorithm with a caliper width of 0.1 standard deviations of the logit of the propensity score. Covariate balance between the matched cohorts was evaluated using standardized mean differences (SMD), with values below 0.1 indicating acceptable balance. In addition, propensity score distributions were visually inspected using density plots to confirm adequate overlap between groups.

### 2.5 Primary and secondary outcomes

The primary outcome was incident HNC, identified using ICD-10-CM diagnostic codes encompassing malignant neoplasms of the lip, oral cavity, pharynx, nasal cavity, middle ear, accessory sinuses, larynx, parotid gland, and other major salivary glands (Supplementary Table 1). Secondary analyses examined three predefined anatomical subtypes: laryngeal cancer, oral cancer (lip, oral cavity, and pharynx), and other HNCs (nasal cavity, paranasal sinuses, and major salivary glands).

To validate our study design, we included positive and negative control outcomes. VDD during follow-up served to confirm the persistence of exposure classification. Osteoporotic fracture, a condition with an established link to VDD, was included as a positive control. Appendicitis, biologically unrelated to vitamin D status, served as a negative control to detect residual confounding. To evaluate potential detection bias, we compared the proportion of patients with documented osteoporosis screening procedure codes between cohorts, serving as a proxy for the intensity of preventive healthcare utilization. All outcomes were assessed from 6 months to 10 years after the index date, with censoring at death or the end of follow-up.

### 2.6 Sensitivity, subgroup, and dose–response analyses

Three sensitivity analyses were conducted to evaluate the robustness of the primary findings. Model I extended the landmark period to 1 year after the index date, excluding patients who developed HNC within the first 12 months to further mitigate reverse causation. Model II applied a 2-year landmark exclusion for the same purpose. Model III addressed the potential selection bias introduced by requiring head and neck–specific diagnostic procedures during follow-up, which could preferentially include patients with a higher pretest probability of cancer detection. In this model, the procedure requirement was removed and replaced with a broader criterion—at least one hemoglobin measurement between 6 months and 10 years after the index date—to confirm ongoing healthcare system contact without restricting the cohort to patients undergoing ENT-specific evaluations.

To explore potential effect modification, pre-specified subgroup analyses were performed, stratifying by sex (female versus male) and age (18–50 years versus >50 years). As an exploratory analysis, we repeated the primary analysis, comparing patients with vitamin D insufficiency (20.0–29.9 ng/mL) against the same control group (≥30.0 ng/mL) to evaluate a potential dose–response relationship between lower vitamin D levels and HNC risk.

### 2.7 Multivariable Cox proportional hazards regression

To further address potential residual confounding by established HNC risk factors, an additional multivariable Cox proportional hazards regression model was constructed for the primary outcome. This model included VDD as the main exposure and adjusted for age at index as a continuous variable, sex, diabetes mellitus, nicotine dependence, liver disease, chronic kidney disease, alcohol-related disorders, and papillomavirus-related disease. This analysis was performed to quantitatively evaluate whether the association between VDD and incident head and neck cancer persisted after accounting for age, sex, HPV-related status, and other clinically relevant covariates.

### 2.8 Statistical analysis

All analyses were performed using the TriNetX Analytics Platform, which provides integrated statistical tools for cohort construction, propensity score matching, and outcome estimation using de-identified electronic health records. The primary measure of association was the hazard ratio (HR) with 95% confidence intervals (CIs), derived from Cox proportional hazards regression. The proportional hazards assumption was evaluated using the Schoenfeld residuals test, with a *p* > 0.05 indicating no significant violation. Kaplan–Meier survival curves were constructed to display the cumulative probability of remaining free of HNC over the 10-year follow-up period, and between-group differences were assessed using the log-rank test. For the primary outcome analysis, statistical significance was defined as a two-sided *p* < 0.05. Secondary, control, sensitivity, and subgroup analyses were considered exploratory; therefore, no adjustment for multiple comparisons was applied, and their results should be interpreted accordingly.

## 3 Results

### 3.1 Patient selection and baseline characteristics

**Figure 1:**
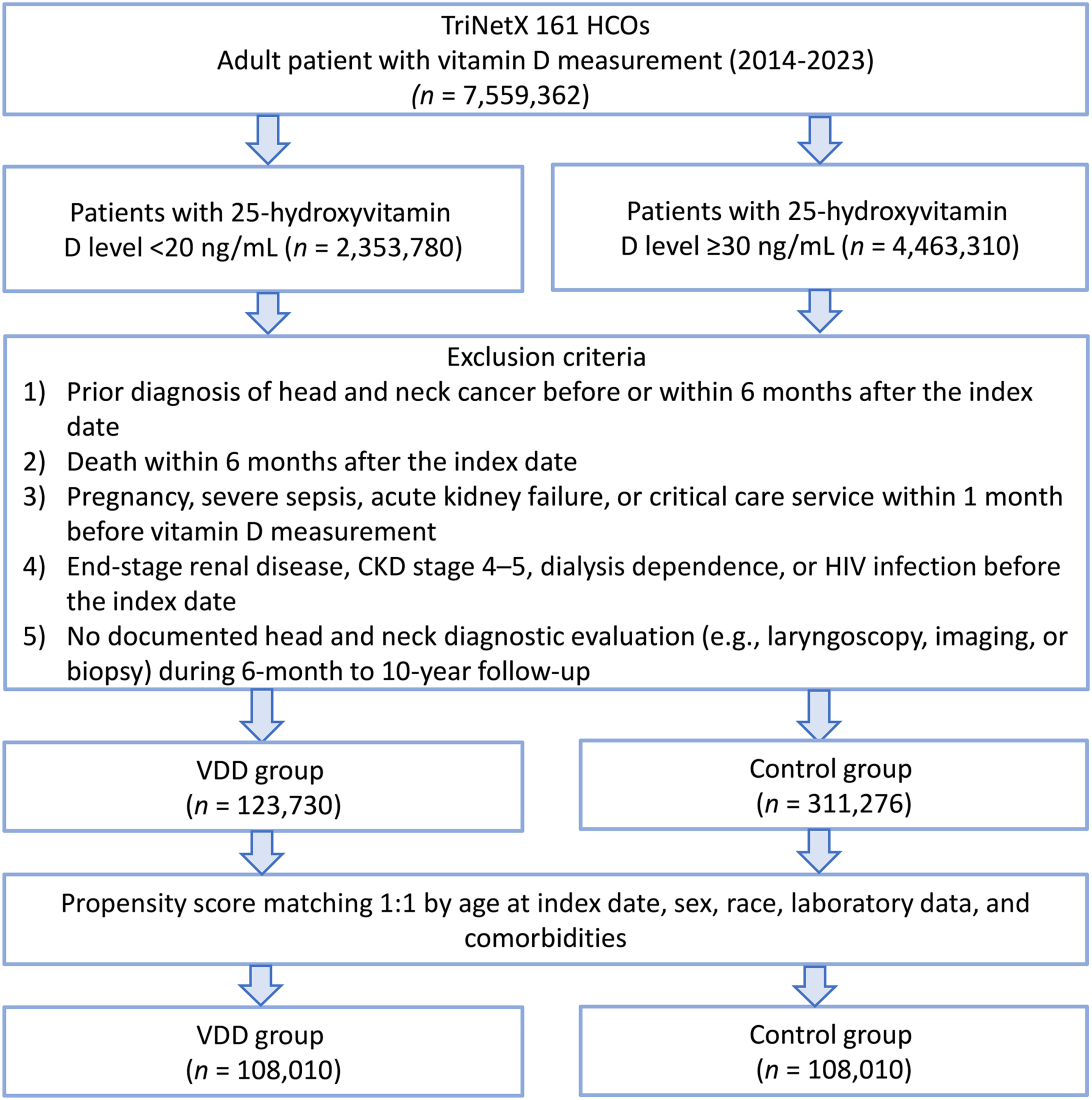
Flowchart of patient selection. Adult patients (≥18 years) with documented serum 25-hydroxyvitamin D measurements between January 2014 and December 2023 were identified from the TriNetX Global Collaborative Network. Patients were classified into the vitamin D deficiency cohort (<20 ng/mL) or the control cohort (≥30 ng/mL). HCOs, healthcare organizations; VDD, vitamin D deficiency; CKD, chronic kidney disease; HIV, human immunodeficiency virus.

**Figure 2:**
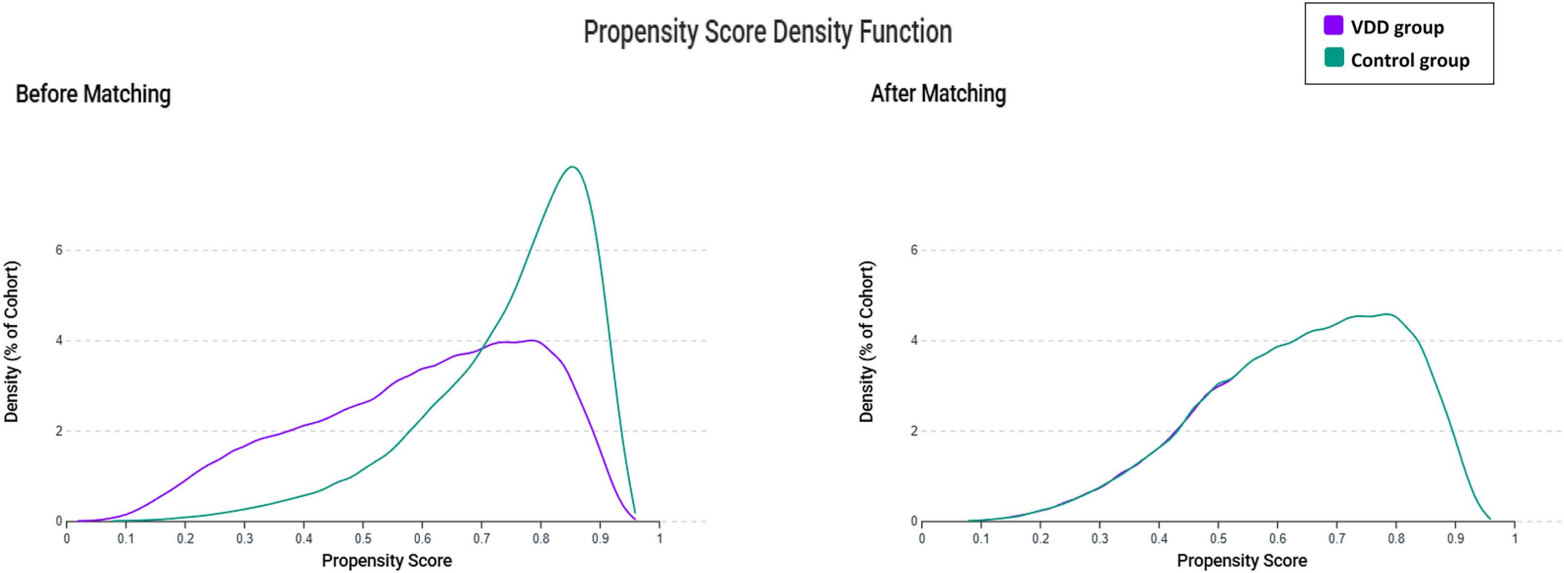
Propensity score density plots before and after matching. Before matching, the vitamin D deficiency group (purple) and the control group (green) showed clearly different propensity score distributions. After 1:1 propensity score matching, the distributions overlapped substantially, indicating adequate covariate balance between groups.

A total of 123,730 patients with VDD and 311,276 control patients met the initial eligibility criteria from the TriNetX Global Collaborative Network (Figure 1). After matching, 108,010 patients remained in the VDD and control cohorts, respectively. Before matching, notable imbalances existed between the groups in terms of age, race, obesity, and nicotine dependence (Table 1). Following 1:1 propensity score matching, 108,010 patients per group were retained, with all standardized mean differences below 0.1, indicating adequate covariate balance (Table 1; Figure 2).

**Table 1:** Baseline characteristics of patients with vitamin D deficiency and controls before and after propensity score matching.

Variables	Before matching		After matching			
	VDD group (*n* = 123,730)	Control group (*n* = 311,276)	SMD†	VDD group (*n* = 108,010)	Control group (*n* = 108,010)	SMD†
Patient characteristics						
Age at index (years)	48.3 ± 16.2	56.7 ± 15.9	0.520	49.9 ± 15.9	50.2 ± 16.4	0.018
BMI ≥ 30 (kg/m^2^)	56,863 (46.0)	103,285 (33.2)	0.264	46,443 (43.0)	47,346 (43.8)	0.017
Female	94,931 (76.7)	248,253 (79.8)	0.073	83,209 (77.0)	82,650 (76.5)	0.012
White	71,724 (58.0)	249,955 (80.3)	0.498	70,656 (65.4)	70,705 (65.5)	0.001
Black or African American	32,797 (26.5)	27,852 (8.9)	0.472	20,457 (18.9)	21,147 (19.6)	0.016
Asian	5,264 (4.3)	12,276 (3.9)	0.016	5,026 (4.7)	4,738 (4.4)	0.013
Factors influencing health status and contact with health services	84,261 (68.1)	213,905 (68.7)	0.013	73,436 (68.0)	72,793 (67.4)	0.013
Comorbidities/medication						
Essential (primary) hypertension	45,986 (37.2)	119,468 (38.4)	0.025	39,618 (36.7)	39,978 (37.0)	0.007
Dyslipidemia	39,149 (31.6)	128,014 (41.1)	0.198	35,871 (33.2)	36,200 (33.5)	0.006
Neoplasms	33,354 (27.0)	104,818 (33.7)	0.147	30,395 (28.1)	29,948 (27.7)	0.009
GERD	30,048 (24.3)	81,752 (26.3)	0.046	26,521 (24.6)	26,600 (24.6)	0.002
Overweight and obesity	33,254 (26.9)	54,043 (17.4)	0.231	25,930 (24.0)	26,113 (24.2)	0.004
Diabetes mellitus	21,114 (17.1)	44,937 (14.4)	0.072	17,605 (16.3)	17,512 (16.2)	0.002
Nicotine dependence	15,663 (12.7)	21,133 (6.8)	0.199	11,710 (10.8)	11,824 (10.9)	0.003
OSA	12,711 (10.3)	28,413 (9.1)	0.039	10,713 (9.9)	10,508 (9.7)	0.006
Anemias	13,144 (10.6)	26,524 (8.5)	0.071	10,454 (9.7)	10,407 (9.6)	0.001
Ischemic heart diseases	9,321 (7.5)	26,328 (8.5)	0.034	8,249 (7.6)	8,053 (7.5)	0.007
Diseases of liver	8,911 (7.2)	19,718 (6.3)	0.035	7,720 (7.1)	7,515 (7.0)	0.007
Family history of primary malignant neoplasm	7,784 (6.3)	24,760 (8.0)	0.065	7,171 (6.6)	6,927 (6.4)	0.009
COPD	6,976 (5.6)	14,745 (4.7)	0.041	5,922 (5.5)	5,942 (5.5)	0.001
Gastritis and duodenitis	5,652 (4.6)	13,241 (4.3)	0.015	4,868 (4.5)	4,738 (4.4)	0.006
Chronic kidney disease (CKD)	4,973 (4.0)	14,572 (4.7)	0.032	4,412 (4.1)	4,270 (4.0)	0.007
COVID-19	3,005 (2.4)	8,269 (2.7)	0.014	2,695 (2.5)	2,646 (2.5)	0.003
Alcohol related disorders	3,229 (2.6)	4,975 (1.6)	0.071	2,432 (2.3)	2,363 (2.2)	0.004
Cerebral infarction	2,209 (1.8)	5,170 (1.7)	0.010	1858 (1.7)	1812 (1.7)	0.003
Malnutrition	1,536 (1.2)	2,845 (0.9)	0.032	1,272 (1.2)	1,228 (1.1)	0.004
Papillomavirus infection	553 (0.4)	1,193 (0.4)	0.010	438 (0.4)	458 (0.4)	0.003
Laboratory data						
Hemoglobin ≥ 12 g/dL	76,823 (62.1)	198,437 (63.8)	0.034	67,540 (62.5)	67,613 (62.6)	0.001
Albumin ≥ 3.5 g/dL	73,932 (59.8)	192,620 (61.9)	0.044	64,777 (60.0)	64,782 (60.0)	0.000
HbA1c ≥ 9%	5,967 (4.8)	6,476 (2.1)	0.151	4,091 (3.8)	4,156 (3.8)	0.003
eGFR ≥ 60	76,085 (61.5)	191,193 (61.4)	0.001	65,755 (60.9)	66,090 (61.2)	0.006
Thyrotropin ≥ 4 m[IU]/L	11,178 (9.0)	29,792 (9.6)	0.018	10,183 (9.4)	9,984 (9.2)	0.006
Medications						
Vitamin D supplementation	17,923 (14.5)	64,302 (20.7)	0.163	16,672 (15.4)	17,145 (15.9)	0.012
Insulins and analogues	10,822 (8.7)	18,296 (5.9)	0.110	8,436 (7.8)	8,370 (7.7)	0.002
GLP-1 analogues	3,401 (2.7)	7,656 (2.5)	0.018	2,930 (2.7)	2,832 (2.6)	0.006
SGLT2 inhibitors	1760 (1.4)	4,306 (1.4)	0.003	1,568 (1.5)	1,570 (1.5)	0.000

Data are presented as *n* (%) or mean ± SD. BMI, body mass index; COPD, chronic obstructive pulmonary disease; eGFR, estimated glomerular filtration rate; GERD, gastroesophageal reflux disease; GLP-1, glucagon-like peptide-1; HbA1c, hemoglobin A1c; OSA, obstructive sleep apnea; SGLT2, sodium-glucose co-transporter 2; SMD, standardized mean difference; VDD, vitamin D deficiency. †SMD values <0.1 indicate adequate balance between groups.

### 3.2 Primary and secondary outcomes

**Figure 3:**
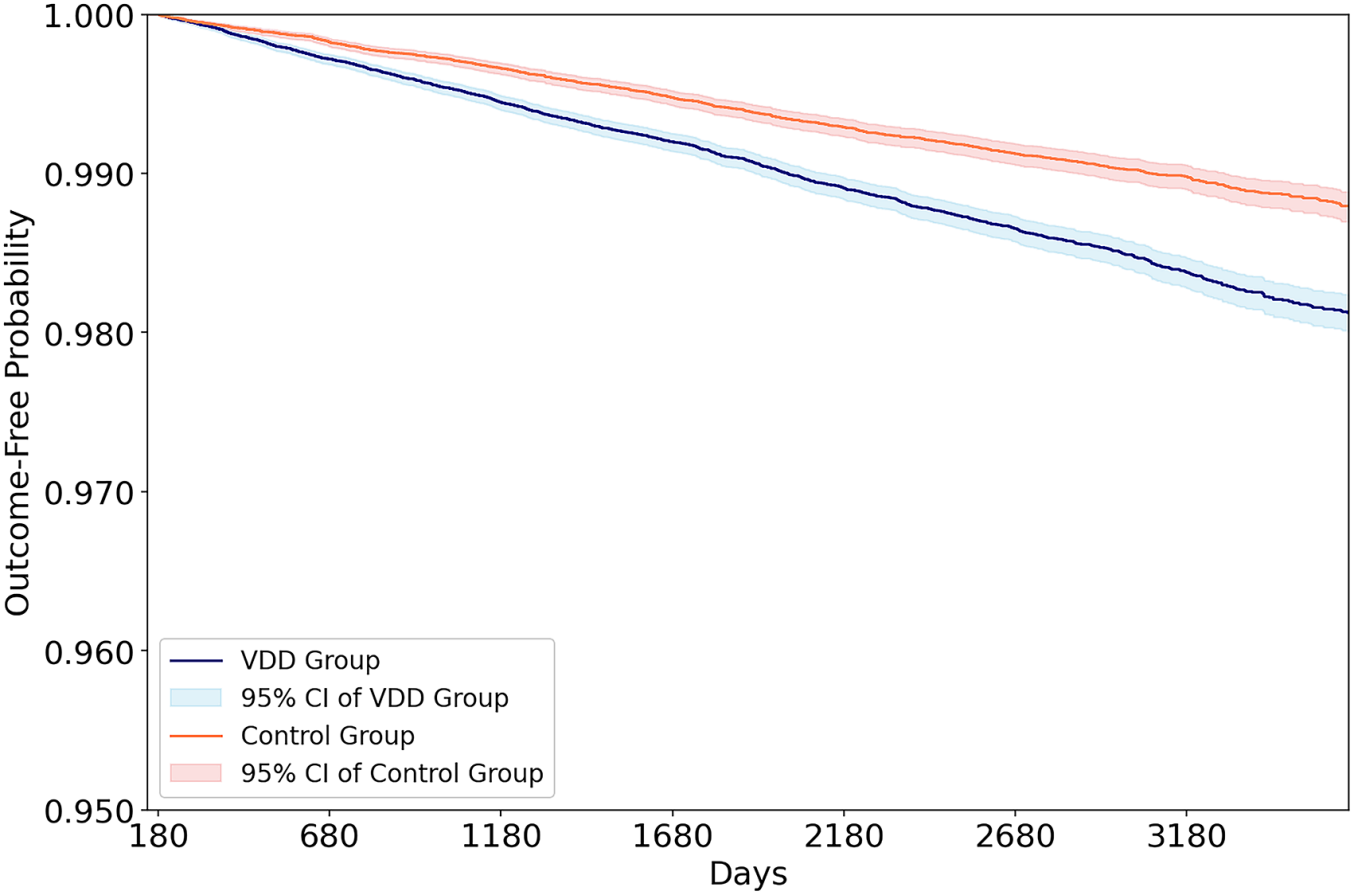
Kaplan–Meier curves for head and neck cancer–free survival over 10 years. The vitamin D deficiency group showed significantly lower cancer-free survival than the control group (log-rank *p* < 0.001). Shaded areas indicate 95% confidence intervals. Follow-up began 180 days after the index date and extended to 3,650 days.

The mean follow-up duration after matching was 6.64 ± 2.59 years in the VDD group and 6.47 ± 2.66 years in the control group. During the observation window of 180 to 3,650 days after the index date, VDD was associated with a significantly higher risk of incident HNC compared to controls (1.22% vs. 0.76%, HR: 1.56, 95% CI: 1.43–1.70, *p* < 0.001) (Table 2; Figure 3). This association was consistent across all three predefined anatomical subtypes, with the strongest magnitude observed for laryngeal cancer (HR: 1.72, *p* < 0.001), followed by oral cancer (HR: 1.53, *p* < 0.001), and other HNCs (HR: 1.46, p < 0.001).

**Table 2:** Association between vitamin D deficiency and head and neck cancer risk during 10-year follow-up.

Outcome	VDD group (*n* = 108,010)	Control group (*n* = 108,010)	HR (95% CI)	*p*-value
	Events (%)	Events (%)		
Primary outcome				
Head and neck cancer	1,318 (1.22)	821 (0.76)	1.56 (1.43–1.70)	<0.001
Secondary outcomes				
Laryngeal cancer	321 (0.30)	182 (0.17)	1.72 (1.43–2.06)	<0.001
Oral cancer	1,057 (0.98)	670 (0.62)	1.53 (1.39–1.69)	<0.001
Other head and neck cancer †	258 (0.24)	172 (0.16)	1.46 (1.20–1.77)	<0.001
Positive control outcomes				
VDD risk	37,040 (34.29)	5,597 (5.18)	8.01 (7.79–8.24)	<0.001
Osteoporotic fracture	1,360 (1.26)	838 (0.78)	1.58 (1.45–1.72)	<0.001
Negative control outcomes				
Appendicitis	1,046 (0.97)	963 (0.89)	1.06 (0.97–1.15)	0.226
Surveillance bias indicator				
Screen for osteoporosis	9,394 (8.70)	9,982 (9.24)	0.91 (0.88–0.94)	<0.001

Data are presented as *n* (%) for events and hazard ratios (HR) with 95% confidence intervals (CI). VDD, vitamin D deficiency. †Includes malignant neoplasms of the nasal cavity, paranasal sinuses, and major salivary glands.

Control outcomes supported the validity of the study design (Table 2). Persistent VDD during follow-up was markedly more prevalent in the VDD group (HR: 8.01, *p* < 0.001), confirming the stability of exposure classification. Osteoporotic fracture, a positive control, was significantly more frequent in the VDD group (HR: 1.58, *p* < 0.001) than in the control group, whereas appendicitis, a negative control, showed no between-group difference (HR: 1.06, *p* = 0.226). Notably, osteoporosis screening rates were slightly lower in the VDD group than in the control group (HR: 0.91, *p* < 0.001), indicating that the VDD group did not receive more intensive medical surveillance and that the observed cancer associations were unlikely attributable to differential detection.

### 3.3 Sensitivity analyses

The association between VDD and HNC remained consistent across all three sensitivity models (Table 3). Extending the landmark exclusion period to one year (Model I; HR: 1.57, *p* < 0.001) or two years (Model II; HR: 1.59, *p* < 0.001) did not materially alter the findings, arguing against reverse causation. When the head and neck diagnostic procedure requirement was replaced with a broader follow-up criterion based on hemoglobin measurement (Model III; HR 1.43, *p* < 0.001), the association was preserved, although slightly attenuated, suggesting that the primary findings were not solely driven by the selection of patients who underwent ENT-specific evaluations. Positive and negative control outcomes behaved as expected across all models.

**Table 3:** Sensitivity analyses of the association between vitamin D deficiency and head and neck cancer risk.

	Model I		Model II		Model III	
Outcomes	HR (95% CI)	*p*-value	HR (95% CI)	*p*-value	HR (95% CI)	*p*-value
Head and neck cancer	1.57 (1.43–1.73)	<0.001	1.59 (1.43–1.76)	<0.001	1.43 (1.29–1.58)	<0.001
Laryngeal cancer	1.77 (1.45–2.17)	<0.001	1.62 (1.29–2.03)	<0.001	1.59 (1.27–1.99)	<0.001
Oral cancer	1.51 (1.36–1.68)	<0.001	1.54 (1.38–1.73)	<0.001	1.40 (1.25–1.56)	<0.001
Other head and neck cancer †	1.45 (1.18–1.79)	<0.001	1.45 (1.15–1.82)	0.001	1.48 (1.19–1.85)	<0.001
VDD risk	6.97 (6.77–7.18)	<0.001	5.86 (5.67–6.05)	<0.001	7.49 (7.39–7.60)	<0.001
Osteoporotic fracture	1.61 (1.47–1.77)	<0.001	1.81 (1.63–2.00)	<0.001	1.38 (1.31–1.45)	<0.001
Appendicitis	1.06 (0.96–1.16)	0.232	1.04 (0.94–1.15)	0.483	1.01 (0.96–1.06)	0.766
Screen for osteoporosis	0.89 (0.86–0.92)	<0.001	0.91 (0.88–0.94)	<0.001	0.87 (0.86–0.89)	<0.001

Data are presented as hazard ratios (HR) with 95% confidence intervals (CI). VDD, vitamin D deficiency. Model I: landmark period extended to 1 year, excluding patients with head and neck cancer diagnosed within 365 days of the index date (*n* = 102,997 per group). Model II: landmark period extended to 2 years, excluding patients with head and neck cancer diagnosed within 730 days of the index date (*n* = 106,215 per group). Model III: head and neck diagnostic procedure requirement removed and replaced with at least one hemoglobin measurement during follow-up as an alternative indicator of ongoing healthcare engagement (*n* = 391,697 per group). †Includes malignant neoplasms of the nasal cavity, paranasal sinuses, and major salivary glands.

### 3.4 Subgroup analyses

Sex-stratified analyses demonstrated a consistent association between VDD and HNC in both females (HR: 1.63, *p* < 0.001) and males (HR 1.43, p < 0.001), with no significant interaction (*p* = 0.150) (Table 4). Age-stratified analyses revealed a significant effect modification (*p* for interaction < 0.001) (Table 5): the association was prominent among patients aged >50 years (HR: 1.58, *p* < 0.001), whereas no significant association was observed in patients aged 18–50 years (HR: 0.91, *p* = 0.507). A similar age-dependent pattern was observed for the positive control outcome of osteoporotic fracture.

**Table 4:** Subgroup analysis by sex: association between vitamin D deficiency and head and neck cancer risk during 10-year follow-up.

Outcomes	Female		Male		*p* for interaction
	HR (95% CI)	*p*-value	HR (95% CI)	*p*-value	
Head and neck cancer	1.63 (1.43–1.87)	<0.001	1.43 (1.28–1.60)	<0.001	0.150
Laryngeal cancer	2.17 (1.58–2.97)	<0.001	1.45 (1.17–1.80)	0.001	0.064
Oral cancer	1.53 (1.32–1.77)	<0.001	1.44 (1.27–1.63)	<0.001	0.540
Other head and neck cancer †	1.36 (1.05–1.76)	0.020	1.45 (1.10–1.90)	0.007	0.742
VDD risk	8.05 (7.80–8.31)	<0.001	7.01 (6.60–7.44)	<0.001	<0.001
Osteoporotic fracture	1.47 (1.35–1.61)	<0.001	1.98 (1.54–2.55)	<0.001	0.055
Appendicitis	0.99 (0.90–1.10)	0.915	1.20 (1.00–1.44)	0.051	0.089
Screen for osteoporosis	0.92 (0.90–0.95)	<0.001	0.76 (0.68–0.85)	<0.001	<0.001

Data are presented as hazard ratios (HR) with 95% confidence intervals (CI). VDD, vitamin D deficiency; NR, not reported due to insufficient events. Female subgroup: *n* = 82,744 per group; male subgroup: *n* = 25,163 per group. †Includes malignant neoplasms of the nasal cavity, paranasal sinuses, and major salivary glands.

**Table 5:** Subgroup analysis by age: association between vitamin D deficiency and head and neck cancer risk during 10-year follow-up.

Outcomes	18–50 years		>50 years		*p* for interaction
	HR (95% CI)	*p*-value	HR (95% CI)	*p*-value	
Head and neck cancer	0.91 (0.67–1.22)	0.507	1.58 (1.45–1.73)	<0.001	<0.001
Laryngeal cancer	NR*	NR*	1.77 (1.47–2.12)	<0.001	NR*
Oral cancer	0.84 (0.61–1.15)	0.275	1.57 (1.42–1.73)	<0.001	<0.001
Other head and neck cancer †	1.30 (0.76–2.22)	0.344	1.45 (1.18–1.77)	<0.001	0.709
VDD risk	6.49 (6.21–6.78)	<0.001	8.88 (8.56–9.21)	<0.001	<0.001
Osteoporotic fracture	0.96 (0.64–1.44)	0.836	1.64 (1.50–1.80)	<0.001	0.002
Appendicitis	1.08 (0.93–1.24)	0.314	0.94 (0.85–1.05)	0.285	0.137
Screen for osteoporosis	0.81 (0.72–0.92)	0.001	0.94 (0.92–0.97)	<0.001	0.013

Data are presented as hazard ratios (HR) with 95% confidence intervals (CI). VDD, vitamin D deficiency; NR, not reported due to insufficient events. Age 18–50 subgroup: *n* = 34,286 per group; age >50 subgroup: *n* = 73,035 per group. †Includes malignant neoplasms of the nasal cavity, paranasal sinuses, and major salivary glands.

### 3.5 Dose–response analysis

To evaluate whether a gradient relationship exists between vitamin D levels and cancer risk, patients with vitamin D insufficiency (20.0–29.9 ng/mL) were compared with controls (≥30 ng/mL) after propensity score matching (*n* = 140,247 per group) (Table 6). Vitamin D insufficiency was associated with a modestly elevated risk of HNC (HR: 1.21, *p* < 0.001), with consistent patterns observed for oral cancer and other HNCs. The association with laryngeal cancer did not reach statistical significance (HR: 1.16, *p* = 0.083). The overall magnitude of association was consistently smaller than that observed in the VDD cohort (HR: 1.56 vs. 1.21), supporting a potential dose–response relationship between lower vitamin D levels and HNC risk.

**Table 6:** Association between vitamin D insufficiency (20.0–29.9 ng/mL) and head and neck cancer risk during 10-year follow-up.

Outcome	VDI group (*n* = 140,247)	Control group (*n* = 140,247)	HR (95% CI)	*p*-value
	Events (%)	Events (%)		
Head and neck cancer	1,553 (1.11)	1,262 (0.90)	1.21 (1.12–1.30)	<0.001
Laryngeal cancer	304 (0.22)	257 (0.18)	1.16 (0.98–1.37)	0.083
Oral cancer	1,280 (0.91)	1,035 (0.74)	1.21 (1.12–1.32)	<0.001
Other head and neck cancer †	371 (0.26)	265 (0.19)	1.37 (1.17–1.60)	<0.001
VDD risk	27,024 (19.27)	9,245 (6.59)	3.14 (3.06–3.21)	<0.001
Osteoporotic fracture	1,670 (1.19)	1,230 (0.88)	1.33 (1.23–1.43)	<0.001
Appendicitis	1,301 (0.93)	1,279 (0.91)	1.00 (0.92–1.08)	0.939
Screen for osteoporosis	12,494 (8.91)	13,005 (9.27)	0.94 (0.92–0.96)	<0.001

Data are presented as *n* (%) for events and hazard ratios (HR) with 95% confidence intervals (CI). VDD, vitamin D deficiency. Outcomes were assessed from 180 to 3,650 days after the index date, with censoring at death or end of follow-up. † Includes malignant neoplasms of the nasal cavity, paranasal sinuses, and major salivary glands.

### 3.6 Multivariable Cox regression analysis

In the multivariable Cox regression analysis, VDD remained independently associated with a higher risk of incident HNC (adjusted HR: 1.52, 95% CI: 1.42–1.62, *p* < 0.001; Table 7). Male sex (HR: 3.90, *p* < 0.001), older age at index (HR: 1.04 per year, *p* < 0.001), nicotine dependence (HR: 2.10, *p* < 0.001), and alcohol-related disorders (HR: 2.32, *p* < 0.001) were also independently associated with HNC risk. In contrast, papillomavirus-related disease was not significantly associated with HNC in this model (HR 0.79, *p* = 0.468), likely reflecting the limited sensitivity of diagnostic coding for HPV-related oncogenic exposure.

**Table 7:** Multivariable analysis.

Variable	HR (95% CI)	*p*-value
VDD vs. control group	1.52 (1.42–1.62)	<0.001
Male	3.90 (3.67–4.15)	<0.001
Age at index	1.04 (1.04–1.05)	<0.001
Diabetes mellitus	0.96 (0.89–1.04)	0.277
Nicotine dependence	2.10 (1.94–2.28)	<0.001
Diseases of liver	1.02 (0.92–1.13)	0.745
Chronic kidney disease	0.98 (0.87–1.09)	0.658
Alcohol-related disorders	2.32 (2.07–2.61)	<0.001
Papillomavirus as the cause of diseases classified elsewhere	0.79 (0.41–1.51)	0.468

Data are presented as adjusted hazard ratios (HRs) with 95% confidence intervals (CIs) from multivariable Cox proportional hazards regression. The model included vitamin D deficiency status, sex, age at index as a continuous variable, diabetes mellitus, nicotine dependence, liver disease, chronic kidney disease, alcohol-related disorders, and papillomavirus-related disease. CI, confidence interval; HR, hazard ratio; VDD, vitamin D deficiency.

## 4 Discussion

In this large, multi-institutional retrospective cohort study of 108,010 propensity score-matched pairs, VDD was associated with a 56% higher risk of incident HNC over a 10-year follow-up period. This association was consistent across all three anatomical subtypes examined, with the strongest magnitude observed for laryngeal cancer. A dose–response pattern was evident, as vitamin D insufficiency (20.0–29.9 ng/mL) was associated with a more modest risk elevation than frank deficiency (<20 ng/mL). The association was consistent across multiple sensitivity analyses and was observed in both sexes, although it was confined to patients older than 50 years. Positive and negative control outcomes behaved as expected, supporting the internal validity of the study design.

To our knowledge, this study represents the largest cohort investigation specifically designed to evaluate the temporal association between prediagnostic vitamin D status and incident HNC risk. Prior prospective studies were limited to fewer than 400 cases and lacked statistical power to examine anatomical subtypes or dose–response gradients (23, 24). Our study addresses these gaps by using a multi-institutional cohort of over 200,000 matched patients with standardized laboratory measurements and a landmark design to mitigate reverse causation. A key finding was evidence of a biological gradient: patients with vitamin D insufficiency (20.0–29.9 ng/mL) demonstrated a 21% increased risk of HNC, whereas those with frank deficiency (<20 ng/mL) demonstrated a 56% increased risk compared to individuals with sufficient levels (≥30 ng/mL). This graded association strengthens the biological plausibility of the observed relationship, as a true etiological factor would be expected to exhibit a monotonic relationship with disease risk. Notably, this dose–response pattern was consistent across oral cancers and other HNCs. The absence of statistical significance for laryngeal cancer in the dose–response analysis may be attributable to the relatively small number of events at this anatomical subsite. Prior studies have also linked vitamin D status or intake with better survival or lower recurrence in patients with established HNC (21, 37, 38). Although these studies address prognosis rather than cancer incidence, they support the broader biological relevance of vitamin D in HNC and provide additional context for our findings.

Our findings should be interpreted in the context of prior prospective studies examining circulating vitamin D and upper aerodigestive tract cancers. Fanidi et al. (23) reported an inverse association between prediagnostic 25(OH)D concentrations and HNC risk, and our results are directionally consistent with this observation. However, the magnitude of association should not be directly compared across studies because of important methodological differences, including exposure modeling, outcome definitions, and statistical measures. Prior cohorts often modeled 25(OH)D as a continuous or log-transformed biomarker and reported odds ratios (23, 24), whereas the present study used clinically defined vitamin D categories and Cox-derived hazard ratios. Therefore, although the observed HR suggests a robust association, it should not be interpreted as evidence that VDD is a causal or among the most potent modifiable risk factors for HNC. Residual confounding from smoking, alcohol use, HPV status, nutrition, socioeconomic factors, and healthcare utilization remains possible despite extensive adjustment and sensitivity analyses.

The consistency of the association across laryngeal, oral, and other HNCs suggests that the relationship between vitamin D status and HNC risk is not confined to a single anatomical subsite. Notably, laryngeal cancer demonstrated the strongest magnitude of association (HR: 1.72), which may reflect site-specific differences in vitamin D metabolism or receptor expression. Although *in vitro* evidence suggests that the vitamin D3 axis in laryngeal cancer cells may be modulated by estrogen receptor status (39), extrapolating these findings to sex-specific population-level associations is speculative. Other unmeasured factors, including household cooking fumes, sexual behaviors, oral HPV infection, and tobacco or alcohol exposure patterns, may also contribute to sex-related differences. Because TriNetX lacks granular behavioral and environmental exposure data, these sex-specific findings should be considered exploratory and hypothesis-generating rather than mechanistic evidence. Oral cancer, which accounted for the largest proportion of events, showed a consistent association across all sensitivity analyses, aligning with prior reports of widespread vitamin D receptor expression in the oral squamous epithelium and the antiproliferative effects of calcitriol on oral cancer cell lines (40–42).

A striking finding was the significant effect of age on this association. The association between VDD and HNC was prominent in patients older than 50 years but absent in those aged 18–50 years. The point estimate below unity observed among adults aged 18–50 years (HR 0.91) was not statistically significant (*p* = 0.507) and should not be interpreted as evidence of a protective effect of VDD. Rather, this null finding may reflect limited statistical power due to fewer outcome events in younger individuals, as well as residual confounding or selection mechanisms. Therefore, this result should be interpreted cautiously and not overemphasized. Several factors may account for this observation. First, the etiology of HNC differs substantially between age groups: younger patients are more likely to harbor HPV-driven tumors, particularly oropharyngeal cancers (43–46), for which the pathogenic pathway may be less influenced by vitamin D–mediated immune surveillance. Second, cumulative exposure to carcinogens, such as tobacco and alcohol, is inherently greater in older populations, and VDD may act as a permissive cofactor that amplifies the oncogenic effects of these established risk factors over prolonged exposure periods. Third, the age-dependent pattern observed for the positive control outcome (i.e., osteoporotic fracture) mirrors that of HNC, suggesting that the biological consequences of VDD become clinically manifest predominantly in older individuals. This finding has potential implications for risk stratification, as it suggests that vitamin D screening and supplementation strategies for cancer prevention may be most relevant in populations aged >50 years. Nevertheless, as this observation is exploratory and hypothesis-generating, it should not be used to inform clinical guidelines or screening recommendations without confirmation from prospective studies.

The incorporation of positive and negative control outcomes strengthens the credibility of our findings. The markedly elevated risk of persistent VDD (HR: 8.01) in the deficiency cohort confirmed that the initial exposure classification remained stable throughout the follow-up period rather than representing a transient laboratory abnormality. Osteoporotic fracture, an outcome with well-established links to VDD, demonstrated the expected positive association (HR: 1.58), providing further evidence that the exposure classification captured a biologically meaningful state. Conversely, appendicitis, a condition with no known biological link to vitamin D, showed no significant difference between the groups (HR: 1.06, *p* = 0.226), arguing against residual confounding as a primary explanation for the observed cancer associations. Additionally, osteoporosis screening rates were slightly lower in the VDD group, indicating that the deficiency cohort did not receive more intensive medical surveillance. This finding mitigates the concern that differential detection could have inflated cancer risk estimates in the VDD group.

The requirement for at least one documented head and neck diagnostic evaluation during follow-up was intended to improve outcome ascertainment, reduce the likelihood of undetected HNC, and ensure continued healthcare system contact, thereby minimizing unrecognized loss to follow-up. Nevertheless, this criterion used post-baseline information and necessarily restricted the primary cohort to adults with clinical indications for ENT-related evaluation, who may differ from the general adult population and may have greater clinical surveillance or otolaryngologic symptoms. Therefore, the primary analysis should be interpreted within this clinically evaluated subgroup. Importantly, this design feature does not constitute classic immortal time bias (47), because vitamin D status was defined entirely at the index date and was not assigned on the basis of any future event. Moreover, when this requirement was removed and replaced with follow-up hemoglobin testing as a broader indicator of healthcare engagement, the association between VDD and HNC remained significant, supporting the robustness of the primary finding beyond this ENT-enriched population.

Several limitations warrant consideration. First, although propensity score matching included proxy measures for key risk factors (e.g., nicotine dependence for smoking, alcohol-related disorders for alcohol use, and papillomavirus infection for HPV status), these diagnostic codes reflect baseline clinical documentation rather than quantitative exposure data (e.g., pack-years, drinks per day) or changes in behavior during follow-up. Residual confounding from imprecisely captured or time-varying lifestyle exposures, therefore, cannot be excluded. Second, the TriNetX platform does not provide access to individual-level patient data or allow users to perform fully customizable multivariate regression analyses, which limits the ability to adjust for potential confounders beyond the variables included in propensity score matching. Third, our 5-year look-back restriction was intended to reduce exposure misclassification, but this strategy may have introduced selection bias. By excluding patients with fluctuating or borderline vitamin D levels, the cohort may have been enriched for individuals with persistently low or consistently sufficient vitamin D status. These stable phenotypes may differ in healthcare utilization, monitoring intensity, or supplementation behavior. Therefore, our findings are most applicable to patients with stable vitamin D status rather than the general population. Fourth, the requirement for head and neck diagnostic procedures during follow-up, while designed to reduce surveillance bias, may have introduced selection toward patients with existing otolaryngologic symptoms. The sensitivity analysis replacing this criterion with hemoglobin measurement yielded consistent results, partially mitigating this concern. Fifth, the study could not distinguish between HNC histological subtypes or differentiate HPV-driven from non-HPV-driven tumors during follow-up, which may have differential relationships with vitamin D status. Finally, as an observational study, causal inference cannot be established, and these findings should be considered hypothesis-generating.

## 5 Conclusion

This multi-institutional retrospective cohort study demonstrated a significant association between VDD and an increased risk of incident HNC, with consistency across anatomical subtypes, a dose–response gradient, and robustness across multiple sensitivity analyses. The association was most prominent in patients older than 50 years, suggesting potential age-dependent susceptibility. Although positive and negative control outcomes supported the internal validity of the study design, the observational nature of the study precluded causal inference. Prospective studies incorporating serial vitamin D measurements, HPV status, and detailed lifestyle data are warranted to confirm these associations and evaluate the potential role of vitamin D optimization in HNC prevention strategies.

## Data Availability

The raw data supporting the conclusions of this article will be made available by the authors, without undue reservation.
