# Supplementary material for: Association between vitamin D deficiency and longitudinal risk of head and neck cancer: a multi-institutional retrospective study
Source: Front Nutr. 2026 Jul 1;13:1826071. doi: 10.3389/fnut.2026.1826071 (PMC13368471; doi:10.3389/fnut.2026.1826071)
Supplement: Supplementary file 1 [file Table_1.DOCX]

**Supplementary Table 1. Codes used for cohort definition, exclusion criteria, outcomes, and matching variables**

| Variable / Definition | Codes |
| --- | --- |
| Inclusion criteria | |
| Adult patients | Age ≥18 years |
| Vitamin D deficiency | TNX:LG25965-1; TNX:9034 (25-hydroxyvitamin D <20 ng/mL) |
| Vitamin D sufficiency (control) | TNX:LG25965-1; TNX:9034 (25-hydroxyvitamin D ≥30 ng/mL) |
| Head and neck diagnostic evaluation | CPT: 31575, 92511, 31231, 76536, 70490, 1010308, 31576, 42800, 41100, 40808, 10005, 10006, 38505 |
| Exposure stability criteria | |
| No prior vitamin D sufficiency (VDD cohort, previous 5 years) | TNX:LG25965-1; TNX:9034 (≥30 ng/mL) |
| No prior vitamin D deficiency (control cohort, previous 5 years) | TNX:LG25965-1; TNX:9034 (<20 ng/mL) |
| Exclusion criteria | |
| Head and neck cancer before or within 6 months after index date | ICD-10-CM: C32, C00–C14, C30, C31, C07, C08 |
| Death before or within 6 months after index date | Demographics: Deceased; ICD-10-CM: R99 |
| End-stage renal disease | ICD-10-CM: N18.6 |
| Advanced chronic kidney disease | ICD-10-CM: N18.4, N18.5 |
| Dialysis dependence | ICD-10-CM: Z99.2 |
| HIV infection | ICD-10-CM: B20, Z21 |
| Pregnancy | ICD-10-PCS: 10; ICD-10-CM: Z33.1; LOINC: 82810-3 |
| Critical care services | CPT: 1013729 |
| Acute kidney failure | ICD-10-CM: N17 |
| Severe sepsis / sepsis | ICD-10-CM: R65.2, A41 |
| Outcome definition | |
| Head and neck cancer | ICD-10-CM: C32, C00–C14, C30, C31, C07, C08 |
| Laryngeal cancer | ICD-10-CM: C32 |
| Oral cancer | ICD-10-CM: C00–C14 |
| Other head and neck cancers (nasal cavity, sinuses, salivary glands) | ICD-10-CM: C30, C31, C07, C08 |
| Vitamin D deficiency (VDD) | TNX:LG25965-1; TNX:9034 (25-hydroxyvitamin D ≤20 ng/mL) |
| Osteoporotic fracture | ICD-10-CM: M80 |
| Screening for osteoporosis | ICD-10-CM: Z13.820 |
| Appendicitis | ICD-10-CM: K35–K38 |
| Variables for propensity score matching | |
| Age | Demographics: Age at index |
| Sex | Female |
| Race | White (2106-3), Black or African American (2054-5), Asian (2028-9) |
| Neoplasms | ICD-10-CM: C00–D49 |
| Overweight and obesity | ICD-10-CM: E66 |
| Diabetes mellitus | ICD-10-CM: E08–E13 |
| Nicotine dependence | ICD-10-CM: F17 |
| Ischemic heart disease | ICD-10-CM: I20–I25 |
| Malnutrition | ICD-10-CM: E40–E46 |
| Other anemia | ICD-10-CM: D64 |
| Liver disease | ICD-10-CM: K70–K77 |
| Dyslipidemia | ICD-10-CM: E78 |
| Cerebral infarction | ICD-10-CM: I63 |
| Obstructive sleep apnea | ICD-10-CM: G47.33 |
| Hypertension | ICD-10-CM: I10 |
| Chronic kidney disease | ICD-10-CM: N18 |
| Family history of malignancy | ICD-10-CM: Z80 |
| Gastroesophageal reflux disease | ICD-10-CM: K21 |
| HPV infection | ICD-10-CM: B97.7 |
| Alcohol-related disorders | ICD-10-CM: F10 |
| Chronic obstructive pulmonary disease | ICD-10-CM: J44 |
| Gastritis and duodenitis | ICD-10-CM: K29 |
| COVID-19 | ICD-10-CM: U07.1 |
| Vitamin D medication | ATC: VT500 |
| GLP-1 analogues | ATC: A10BJ |
| SGLT2 inhibitors | ATC: A10BK |
| Insulins and analogues | ATC: A10A |
| Body mass index | TriNetX: 9083 |
| Albumin | TriNetX: 9045 |
| Hemoglobin | TriNetX: 9014 |
| Hemoglobin A1c | TriNetX: 9037 |
| Estimated glomerular filtration rate | LOINC: 62238-1 |
| Thyrotropin | TriNetX: 9040 |
